# Catatonia in adult anti-NMDAR encephalitis: an observational cohort study

**DOI:** 10.1186/s12888-022-04505-x

**Published:** 2023-02-07

**Authors:** Huiting Wu, Chunmei Wu, Yingying Zhou, Shanshan Huang, Suiqiang Zhu

**Affiliations:** Department of Neurology, Tongji Hospital, Tongji Medical College, Huazhong University of Science and Technology, 1095 Jiefang Avenue, Wuhan, 430030 Hubei China

**Keywords:** Anti-NMDAR encephalitis, Catatonia, Outcome

## Abstract

**Background:**

Anti-N-methyl-D-aspartate receptor (NMDAR) encephalitis is one of the most prevalent autoimmune encephalitis and is closely related to catatonia. This study aimed to investigate the clinical features and disease outcomes of adult catatonic anti-NMDAR encephalitis patients.

**Methods:**

Adult patients diagnosed with anti-NMDAR encephalitis between January 2013 and October 2021 were retrospectively enrolled in this study. According to the Bush Francis Catatonia screening instrument (BFCSI), patients were divided into two groups: those with catatonia and those without catatonia. The modified Rankin scale (mRS), Clinical Assessment Scale for Autoimmune Encephalitis (CASE), Neuropsychiatric Inventory (NPI), Patient Health Questionnaire-9 (PHQ-9) and 7-item Generalized Anxiety Disorder Questionnaire (GAD-7) scores were assessed at follow-up. The Mann–Whitney U test (nonparametric), Student’s t test (parametric), and chi-squared test were used to analyse the differences between the two groups.

**Results:**

Eighty-four patients were recruited, including twenty-five catatonic patients and fifty-nine noncatatonic patients. Among them, 28 had positive antibody only in cerebrospinal fluid (CSF), 4 had positive antibody only in serum and 52 had positive antibody both in CSF and serum. Catatonic patients experienced more disturbance of consciousness (*p* = 0.01), aggression (*p* = 0.046) and affective disorders (*p* = 0.043) than noncatatonic patients. The mRS scores of the catatonia group assessed at admission (*p* = 0.045) were worse than those of the non-catatonia group. Catatonic patients were more inclined to develop deep vein thrombosis (*p* = 0.003), decubitus (*p* = 0.046), pneumonia (*p* = 0.025), and to be admitted to the intensive care unit (ICU) (*p* = 0.011) than noncatatonic patients. All patients in the catatonia group received first-line immunotherapy. At the 24-month follow-up, 2 patients in the catatonia group did not achieve good outcomes. At the last follow-up, the catatonia group had more relapses (*p* = 0.014) and more neuropsychiatric problems (*p* = 0.035).

**Conclusions:**

Adult anti-NMDAR encephalitis patients with catatonia present distinct clinical features in disease course and are prone to experience more relapses and long-term neuropsychiatric problems than those without catatonia.

**Supplementary Information:**

The online version contains supplementary material available at 10.1186/s12888-022-04505-x.

## Introduction

Catatonia, a subtype of schizophrenia in the past, is a psychomotor syndrome consisting of several symptoms, such as stupor, mutism, and agitation. However, according to the Diagnostic and Statistical Manual of Mental Disorders, Fifth Edition (DSM-5) [1], the catatonic subtype of schizophrenia has been eliminated and catatonia can be diagnosed not only as an episode specifier for schizophrenia, but also as a specifier for major mood disorders and several other psychotic disorders. It can also be diagnosed as secondary to general medical condition and as unspecified category without clear recognition of underlying causes. In the 11th revision of the International Classification of Diseases (ICD-11), new diagnostic grouping has been added and catatonia can be divided into three conditions: (1) associated with another mental disorder; (2) induced by psychoactive substances, including medications; and (3) secondary catatonia due to a medical condition [2]. These changes made catatonia no longer limited to schizophrenia, but become an independent nosological entity, facilitating the identification of catatonia in the context of other psychotic disorders, the use of substances and general medical conditions, such as encephalitis [3, 4]. Several studies revealed that serious medical complications might occur if catatonia cannot be recognized and interventions are not initiated in a timely manner [5, 6]. With improper management, catatonia can be aggravated to malignant forms, namely neuroleptic malignant syndrome (NMS) or malignant catatonia (MC), usually presenting as hyperpyrexia and severe autonomic dysfunction or muscle rigidity with lethal potential [7, 8]. It is of great importance to recognize catatonia and signs of deterioration in patients with diseases with life-threatening conditions.

Anti-N-methyl-D-aspartate receptor (NMDAR) encephalitis, mediated by antibodies against the GluN1 subunit of the NMDAR [9], is one of the most common types of autoimmune encephalitis and characterized by psychiatric symptoms, cognitive impairment, seizures, abnormal movements, autonomic dysfunction or a decreased level of consciousness. Catatonia is closely associated with anti-NMDAR encephalitis [10]. In a systematic review of all cases of catatonia in autoimmune diseases, anti-NMDAR encephalitis accounted for 72% of cases [11]. Although this proportion is large, only a few studies have focused on the description of catatonic syndrome in anti-NMDAR encephalitis [12–14]. In addition, autoimmune disease-related catatonia was found to be associated with other severe psychiatric features and more relapses in children [15]. Etiological treatment is fundamental for the treatment of catatonia secondary to various disorders. And benzodiazepines are recommended as the first-line symptomatic medication for catatonia. Electroconvulsive therapy (ECT) is an alternative treatment when benzodiazepines are ineffective or malignant situations occur. Other management strategies have also been proposed [16, 17]. The specific symptomatic therapies showed various efficacy in different populations and etiologies, and the treatment of etiology is sufficient or even more important than the specific one, for example in the elderly, schizophrenia spectrum disorders, obsessive-compulsive disorder, or in different cases of organic causes such as anti-NMDAR encephalitis [14, 18–20]. Although the treatment strategy for catatonia in anti-NMDAR encephalitis has not been well formulated, immunotherapy usually brought clinical improvement for catatonia in anti-NMDAR encephalitis [12]. The administration of antipsychotics is prevalent in anti-NMDAR encephalitis due to the high morbidity of psychiatric symptoms. Although in a few cases relief from agitation and psychotic symptoms is achieved in patients with catatonia, antipsychotic drugs can aggravate the syndrome and lead to malignant forms [21]. To date, research on the clinical features and prognosis of patients with catatonia in anti-NMDAR encephalitis is still lacking.

Therefore, the purposes of this study are as follows: (1) to describe the frequency, characteristics, treatment and clinical outcomes of catatonia in adults with anti-NMDAR encephalitis and (2) to explore the occurrence of severe complications and the malignant forms of catatonia in patients with anti-NMDAR encephalitis.

## Methods and materials

### Participants

For this single-centre retrospective observational cohort study, we consecutively enrolled inpatients definitively diagnosed with anti-NMDAR encephalitis, according to the criteria proposed by Graus et al. [22], at the Neurology Department of Tongji Hospital from January 2013 to October 2021. Antibodies were detected in serum or cerebrospinal fluid (CSF) by a commercial cell-based assay (CBA) before the initiation of immunotherapy and if only serum was available, antibody may also be tested by immunohistochemistry of rat brain. Patients who met the following criteria were excluded: (1) patients who could be reasonably diagnosed with other central nervous system disorders mimicking anti-NMDAR encephalitis; (2) patients with incomplete medical records; and (3) patients under the age of 18 years.

The study was approved by the Ethics Committee of Tongji Hospital, Tongji Medical College of Huazhong University of Science and Technology. Informed consent was obtained from all patients or their representatives when the patients were unable to understand and provide valid consent. The anonymity of patients was preserved in the study.

### Catatonia assessment

All patients were screened by the 14-item Bush-Francis Catatonia Screening Instrument (BFCSI) [23], the most widely used catatonia rating scale, given its excellent reliability and validity [24, 25]. When patients showed two or more out of the following 14 signs: excitement, immobility, mutism, staring, posturing, grimacing, echopraxia/echolalia, stereotypy, mannerisms, verbigeration, rigidity, negativism, waxy flexibility and withdrawal, they were assigned to the catatonia group. According to the symptoms present, catatonia was also divided into agitated, withdrawn or fluctuating types.

MC and NMS were both viewed as malignant forms of catatonia in this study [8, 26–30]. Patients with MC were defined as those with catatonia developing severe autonomic disorders (tachycardia, elevated/instable blood pressure, diaphoresis, and tachypnoea) and fever (body temperature ≥ 38.0 °C) [31]. According to the DSM-IV criteria, patients with NMS were characterized by severe muscle rigidity and elevated temperature and the additional presence of two or more of the following characteristic features: altered blood pressure, altered consciousness, diaphoresis, dysphagia, incontinence, laboratory evidence of muscle injury, leucocytosis, mutism, rigor, tachycardia and tremor.

### Clinical variables and outcomes

The medical records of each patient were checked separately by 3 neurologists, and any discrepancies were discussed. The following information was collected: age, sex, duration of hospitalization, antibody titres in serum and CSF, major neuropsychiatric manifestations (psychotic symptoms, cognitive impairment, speech disturbance, seizures, movement disorder, change in consciousness and autonomic disturbance), presence and type of catatonia, presence of malignant forms of catatonia, presence of neoplasm, complications, immunotherapy received, psychiatric treatments prescribed (benzodiazepines and antipsychotics). We also obtained and recorded abnormal findings on magnetic resonance imaging (MRI), electroencephalograpy (EEG) and CSF examinations. CSF was initially tested at a 1:1 dilution, and serum was tested at a 1:10 dilution. Antibody titres of 1:10 in serum or 1:1 in CSF were considered low, ≤ 1:100 in serum or ≤ 1:10 in CSF were considered moderate, and ≥ 1:320 in serum or ≥ 1:32 in CSF were considered high. In addition to catatonia, psychotic symptoms were subdivided into abnormal behaviour, psychosis (delusion or hallucination), mood (anxiety, depression, irritability and so on), sleep, suicidality, eating and obsessive-compulsive disorders [32]. First-line immunotherapy included corticosteroids, intravenous immunoglobulin and plasmapheresis. Second-line immunotherapy consisted of rituximab, cyclophosphamide and mycophenolate mofetil. The order of treatments administered or their combination within or between each line of treatments was not specified.

The Clinical Assessment Scale in Autoimmune Encephalitis (CASE) [33] is a novel severity scale for autoimmune encephalitis with good reliability and validity. To compare the outcomes at different courses, we evaluated both the modified Rankin scale (mRS) and CASE score at onset and at months 6, 12, and 24. Poor outcomes were defined as an mRS score ≥ 3 at follow-up. Unfavourable clinical outcomes were also defined by a CASE score ≥ 4 [34]. Relapse was identified as the presence of any new symptoms or a worsening of previous symptoms after being stable for 2 months. At the last follow-up, the Neuropsychiatric Inventory (NPI), the Patient Health Questionnaire-9 (PHQ-9) and the 7-item Generalized Anxiety Disorder Questionnaire (GAD-7) were administered to assess the long-term neuropsychiatric outcomes.

### Statistical analysis

Data analysis was performed by SPSS software (version 26). Quantitative variables with a normal distribution are shown as the mean ± SD, and Student’s t test was applied for intergroup comparisons. Continuous variables with nonnormal distributions are presented as medians with interquartile ranges (IQRs) and were compared using the Mann–Whitney U test. Categorical variables are presented as frequencies (percentages) and were compared by either the chi-square test or Fisher’s exact test. Relapse frequency was analysed by Kaplan–Meier curves with log-rank statistics. A value of p less than 0.05 was considered significant.

## Results

### General findings

**Fig. 1:**
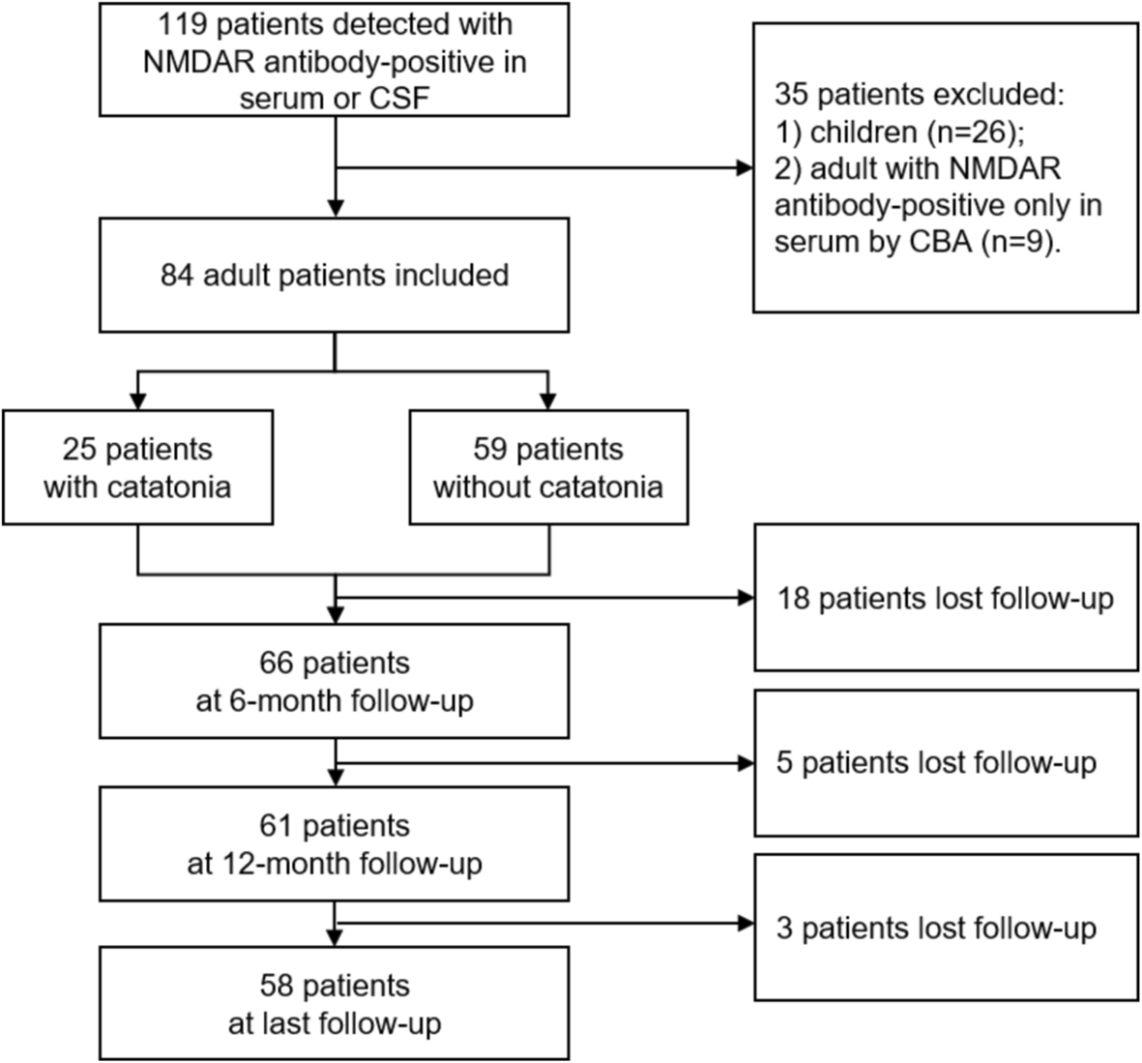
Flow chart of study patients

This study initially included 84 adult patients with anti-NMDAR encephalitis (Fig. 1). Eighty samples from CSF and 63 samples from serum were obtained. Among them, 28 had positive antibody only in CSF, 4 had positive antibody only in serum, and 52 had positive antibody both in CSF and serum. Twenty-six paediatric patients were excluded. Table 1 shows the main characteristics of our patients. Of the 84 patients, 53.6% were female and 46.4% were male. The median age was 29.0 (21.0-43.0) years. The median duration of hospitalization was 19.0 (13.0-31.0) days.

**Table 1:** Characteristics and treatment in catatonia group and non-catatonia group with anti-NMDAR encephalitis

Variables	All	Non-catatonia	Catatonia	***p***-value^*^
Age, median (IQRs)	29.0(21.0-43.0)	29.0(21.0-44.3)	32.0(22.0-42.0)	0.761
Female, n (%)	45(53.6)	32(54.2)	13(52.0)	0.851
Duration of hospitalization, median (IQRs)	19.0(13.0-31.0)	17.5(13.0-27.3)	25.0(13.0-43.5)	0.233
mRS in admission	3(2-4)	3(1-4)	3(2-4.5)	0.045
Auxiliary examination				
D-Dimer (*n* = 78) (mg/L) (IQRs)	0(0-1.5)	0(0-1.0)	0(0-2.7)	0.289
Leucocytosis in CSF	60(71.4)	42(71.2)	18(72.0)	0.94
Abnormal EEG^a^ (*n* = 51), n (%)	36(70.6)	26(70.3)	10(71.4)	> 0.999
Abnormal MRI^b^ (*n* = 78), n (%)	45(57.7)	28(50.9)	17(73.9)	0.061
Psychiatric history, n (%)	1(1.2)	0(0)	1(4.0)	0.298
Symptomatology, n (%)				
Prodrome	54(64.3)	38(64.4)	16(64.0)	0.972
Psychiatric symptoms initially	33(39.3)	22(37.3)	11(44.0)	0.565
Cognitive impairment	50(59.5)	35(59.3)	15(60.0)	0.954
Speech disturbance	36(42.9)	18(30.5)	18(72.0)	< 0.001
Movement disorder	21(25.0)	14(23.7)	7(28.0)	0.679
Disturbance of consciousness	39(46.4)	22(37.3)	17(68.0)	0.01
Autonomic disturbance	19(22.6)	12(20.3)	7(28.0)	0.443
Seizures	56(66.7)	37(62.7)	19(76.0)	0.238
Status epilepticus	18(21.4)	11(18.6)	7(28.0)	0.339
Psychiatric symptoms	68(81.0)	43(72.9)	25(100.0)	0.01
Behavior	60(71.4)	37(62.7)	23(92.0)	0.007
Aggression	12(14.3)	5(8.5)	7(28.0)	0.046
Psychosis	19(22.6)	13(22.0)	6(24.0)	0.844
Hallucination	18(21.4)	13(22.0)	5(20.0)	0.835
Delusion	5(6.0)	2(3.4)	3(12.0)	0.307
Mood	30(35.7)	17(28.8)	13(52.0)	0.043
Irritability	7(8.3)	3(5.1)	4(16.0)	0.221
Anxiety	9(10.7)	5(8.5)	4(16.0)	0.526
Depression	10(11.9)	7(11.9)	3(12.0)	> 0.999
Sleep disorder	35(41.7)	22(37.3)	13(52.0)	0.211
Suicidality	3(3.6)	3(5.1)	0(0)	0.551
Eating	5(6.0)	3(5.1)	2(8.0)	0.99
Delirium	8(9.5)	5(8.5)	3(12.0)	0.923
Admission to ICU, n (%)	33(39.3)	18(30.5)	15(60.0)	0.011
Mechanical ventilation, n (%)	18(21.4)	11(18.6)	7(28.0)	0.339
Comorbidities and complications, n (%)				
Tumour	4(4.8)	3(5.1)	1(4.0)	> 0.999
Other immune diseases	3(3.6)	3(5.1)	0(0)	0.551
Decubitus	12(14.3)	5(8.5)	7(28.0)	0.046
Deep vein thrombosis	7(8.3)	1(1.7)	6(24.0)	0.003
Acute kidney failure	3(3.6)	2(3.4)	1(4.0)	> 0.999
Pneumonia	38(45.2)	22(37.3)	16(64.0)	0.025
Immunotherapy, n (%)				
First-line	80(95.2)	55(93.2)	25(100.0)	0.439
Corticosteroids	79(94.0)	54(91.5)	25(100.0)	0.319
Gamma globulin	32(38.1)	19(32.2)	13(52.0)	0.088
Plasmapheresis	7(8.3)	4(6.8)	3(12.0)	0.719
Second-line	5(6.0)	3(5.1)	2(8.0)	0.990
Symptomatic treatment				
Atypical antipsychotics	57(67.9)	34(57.6)	23(92.0)	0.002

^*^P-value less than 0.05 is of significance

^a^Abnormal EEG was defined as the presence of focal or diffuse slow or disorganized activity, epileptic activity, or extreme delta brush

^b^Abnormal MRI was defined as hyperintense signal on T2- weighted or fluid- attenuated inversion recovery sequences in medial temporal lobes, or in multifocal areas involving grey matter, white matter, or both compatible with demyelination or inflammation

The most prevalent manifestations in anti-NMDAR encephalitis according to frequency were as follows: psychiatric symptoms (81.0%), seizures (66.7%), cognitive dysfunction (59.5%), decreased level of consciousness (46.4%), speech disorder (42.9%), movement disorder (25.0%), and autonomic disturbance (22.6%). Thirty-three (39.3%) patients initially presented with psychiatric abnormalities. The proportion of patients with leucocytosis in CSF and abnormal EEG and MRI examinations was 71.4% (60/84), 70.6% (36/51) and 55.1% (43/78), respectively. Of the patients with EEG records, 31.4% (16/51) were with diffuse slow-wave activity, 21.6% (11/51) were with epileptiform discharges, 5.9% (3/51) were with extreme delta brush, 3.9% (2/51) were with focal slow-wave activity, and the remaining abnormal records (19.6%, 10/51) lacked detailed description, thus it was difficult to further analyse the significance of abnormal waveform like extreme delta brush in clinical. Of the patients with MRI records, 35.9% (28/78) had multifocal lesions, 16.7% (13/78) had lesions on temporal lobe, 5.1% (4/78) had lesions on other lobes. Three patients were found to have ovarian teratomas, and uterine leiomyoma was detected in one patient. A total of 35.0% (28/80) and 19.0% (12/63) of the patients had high antibody titres in CSF and serum, respectively. At admission, the median mRS score was 3. Overall, 95.2% (80/84), 6.0% (5/84) and 67.9% (57/84) of patients received first-line immunotherapy, second-line immunotherapy and antipsychotic drugs, respectively (Table 1).

At the 24-month follow-up, the median mRS score of 58 patients was 0 (0-2), and the median CASE score was 1 (1-3). A total of 15.5% of patients had poor outcomes according to mRS scores, and 19.0% had unfavourable outcomes according to CASE scores (Additional file 1: Supplementary Table S1). We kept contact with those patients to assess their long-term psychiatric outcomes. The mortality rate in this study was 7.1% (6/84) and 6 patients all died within 6 months of follow-up. In non-catatonic group, two patients died of severe pneumonia, two patients died of unclear causes and one patient was suspected to die of cerebral venous thrombosis. One catatonic patient died of multiple organ failure. Fifty-two patients were evaluated by the scales mentioned previously at the last follow-up (Table 2). The median time from discharge to the latest follow-up was 46 (30-76) months.

**Table 2:** Outcomes of patients with anti-NMDAR encephalitis between two groups at the last follow-up

Outcomes	All	Non-catatonia	Catatonia	***p***-value^*^
Death, n (%) (*n* = 58)	6(10.3)	5(13.5)	1(4.8)	0.546
Relapse, n (%) (*n* = 58)	9(15.5)	2(5.4)	7(33.3)	0.014
Neuropsychiatric symptoms, n (%) (NPI ≥ 1, *n* = 52)	17(32.7)	7(21.9)	10(50.0)	0.035
Anxiety, n (%) (GAD-7 ≥ 5, *n* = 52)	6(11.5)	1(3.1)	5(25.0)	0.05
Depression, n (%) (PHQ-9 ≥ 5, *n* = 52)	5(9.6)	2(6.3)	3(15.0)	0.577

^*^P-value value less than 0.05 is of significance

### Catatonia group

**Fig. 2:**
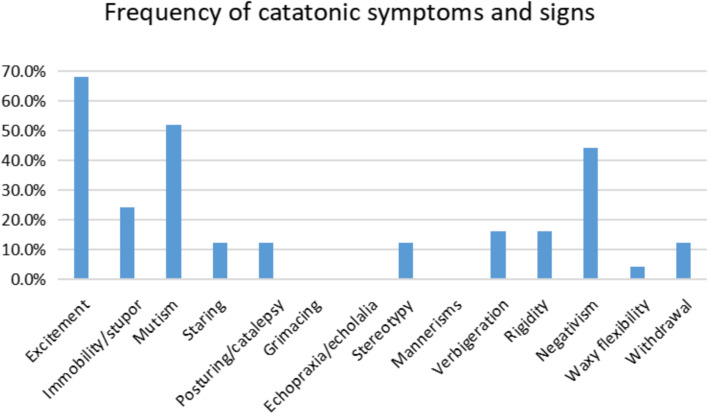
Frequency of catatonic signs and symptoms in catatonia group with anti-NMDAR encephalitis

Twenty-three patients were defined with catatonia according to BFCSI. Sixteen (64.0%) patients had a history of prodrome. Regarding the psychopathology of catatonic patients, 92.0% presented with abnormal behaviours, 24.0% presented with psychosis symptoms such as hallucinations and delusions, 52.0% exhibited mood disorders, 52.0% had sleep disturbances and 8.0% showed eating disorders (Table 1). One patient was diagnosed with ovarian teratoma. The most common signs of catatonia syndrome were excitement (68.0%), mutism (52.0%), negativism (44.0%) and immobility/stupor (24.0%). Symptoms such as echopraxia/echolalia, grimacing and mannerism were not found in medical records (Fig. 2). In the catatonia group, 28.0, 40.0, and 32.0% of patients were diagnosed with the agitated, withdrawn and fluctuating type of catatonia, respectively.

Patients in the catatonia group were more likely to have disturbance of consciousness (*p* = 0.01), aggression (*p* = 0.046) and mood disorders (*p* = 0.043) than those in the non-catatonia group (Table 1). The median mRS score assessed at onset was worse in the catatonia group (*p* = 0.045). There was no significant difference in antibody titres detected in serum (*p* = 0.864) or CSF (*p* = 0.971) between two groups.

All patients with catatonia received first-line immunotherapy. Thirteen (52.0%) patients received intravenous injection of gamma globulin, and three (12.0%) patients underwent plasmapheresis. Two (8.0%) patients received second-line immunotherapy. Antipsychotics were administered to 23 (92.0%) patients (*p* = 0.002). Benzodiazepines were used in 17 (68.0%) patients. The difference in the proportion of patients receiving first-line and second-line therapies did not reach significance (Table 1).

During the disease course, malignant forms of catatonia were recorded in 4 (16.0%) patients. All four of these patients suffered from significant hypotension. One of them had severe tachycardia, tachypnoea, sialorrhea and rigidity.

According to the findings of a detailed search, the occurrence rates of deep vein thrombosis, decubitus, pneumonia and acute kidney failure in the catatonia group were 24.0, 28.0, 64.0, and 4.0%, respectively. Catatonic patients were more likely to develop deep vein thrombosis (*p* = 0.003), decubitus (*p* = 0.046), pneumonia (*p* = 0.025), and to be admitted to the intensive care unit (ICU) (*p* = 0.011) (Table 1).

**Fig. 3:**
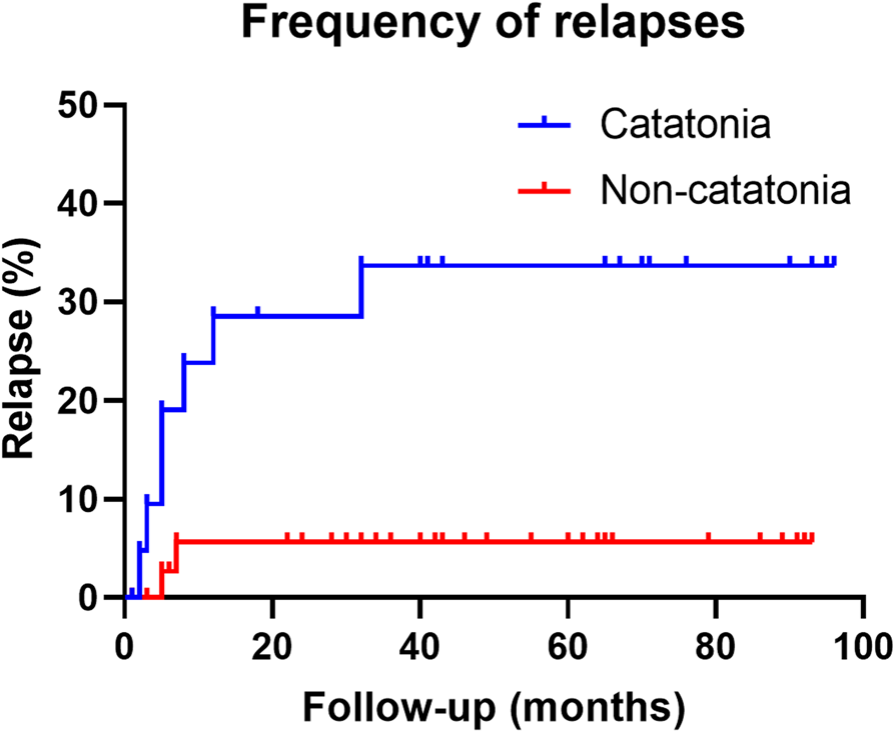
Frequency of relapses in catatonia group and non-catatonia group

At the 24-month follow-up, the long-term outcomes as determined by the mRS (*p* = 0.551) and CASE (*p* = 0.929) were not different between the groups with and without catatonia. Catatonic patients were more likely to suffer from relapses (*p* = 0.014) and neuropsychiatric problems (*p* = 0.035), and had a tendency to develop anxiety (*p* = 0.05) at the last follow-up (Table 2). The curve showing the relapse frequency of patients in different groups (*p* < 0.05) is presented in Fig. 3.

## Discussion

Catatonia in anti-NMDAR encephalitis has drawn increasing attention, and this is the first study focusing on the long-term prognosis of adult patients with anti-NMDAR encephalitis and catatonia. In our study, we observed that (1) patients in the catatonia group tended to have more neuropsychiatric and medical complications during the acute stage; (2) the majority of patients with catatonia achieved favourable outcomes with use of immunotherapy; and (3) the presence of catatonia might serve as a predictor of more relapses and long-term neuropsychiatric problems. Considering the scarcity of research, our study provides a comprehensive view of catatonia in anti-NMDAR encephalitis.

Anti-NMDAR encephalitis predominates as the cause of autoimmune-related catatonia, accounting for 72% (249/346) of cases [11]. In two retrospective clinical studies, the incidence of catatonia in anti-NMDAR encephalitis was 13.9% (15/108) and 47% (14/30), respectively [35, 36]. And the rate was reported at 32.7% in a systematic literature review of 633 cases diagnosed with anti-NMDAR encephalitis [37]. Consistent with these findings, our study showed that the rate of catatonia in anti-NMDAR encephalitis was 29.8% (25/84), demonstrating the common catatonic manifestations in anti-NMDAR encephalitis. However, with multiple rating scales and improved criteria, the incidence rate was up to 70.6% in a prospective study recruiting 58 anti-NMDAR encephalitis [12]. It was even much higher (83.3%, 10/12) in a prospective study enrolling patients initially diagnosed with schizophrenia, mood disorder, or epilepsy with psychiatric symptoms [38]. It was also reported in another prospective research that four NMDAR antibody-positive patients whose initial diagnosis was mood disorder all showed catatonic symptoms [39]. The gap of prevalence between prospective and retrospective studies brought out the significance of consistent diagnostic protocols and systematic application of psychopathological measures, which could otherwise cause an underestimation or misdiagnosis of catatonia, especially for secondary catatonia. This may also explain the discrepancy of catatonic signs and symptoms, and accompanied neuropsychiatric symptoms between studies, as mentioned below.

The spectrum of clinical features of catatonia in anti-NMDAR encephalitis still needs to be explored. A review including 189 patients showed that the most frequent signs were immobility/stupor, mutism, excitement, posturing/catalepsy, stereotypy and rigidity [13]. In another review involving 30 cases, excitement, immobility/stupor, mutism, withdrawal, posturing, rigidity, stereotypy and perseveration were successively the most common catatonic symptoms [40]. Our study found that the most frequent catatonic signs in descending order were excitement, mutism, negativism and immobility/stupor, while symptoms such as echopraxia/echolalia, grimacing and mannerisms did not appear in the records. In a prospective study, Espinola-Nadurille et al. observed that the most prevalent catatonic symptoms were immobility, staring, mutism and posturing [12], which not only covered the results of the retrospective studies, but also reported additional common symptoms. Despite the limited number of patients, this discrepancy in signs and symptoms supported the heterogeneous presentations of catatonia in anti-NMDAR encephalitis. Moreover, the lack of consistent operational definitions and systematic division of psychopathology may also make the discrepancy. The stuporous type and the excited type are two classic types of catatonia [13]; however, approximately 33 to 56.1% of catatonic patients with anti-NMDAR encephalitis presented with fluctuations between stuporous and excited symptoms [12, 13, 40]. The fluctuating type was found in a large proportion of patients in our cohort (32.0%), replicating this unique phenomenon in catatonia with anti-NMDAR encephalitis.

Catatonia in anti-NMDAR encephalitis is likely to be accompanied by several neuropsychiatric symptoms. Espinola-Nadurille et al. reported catatonia with more delirium, hallucinations and psychomotor agitation during hospitalization [12]. Serra-Mestres et al. reported that catatonia was frequently combined with dysfunctions of psychosis, mood (anxiety, elation, or depression) and cognition [13]. In our study, we found that symptoms tended to occur together with signs such as aggression, mood disorder and decreased consciousness. The coexistence of those symptoms implied the complexity of the disease and well supported the wide use of psychiatric drugs in catatonia. Nevertheless, clinicians should be cautious about the risk of aggravating catatonia with drug management [21]. The proportion of malignant forms of catatonia caused by anti-NMDAR encephalitis was significantly higher than that due to other causes, with up to 46.7% of patients receiving antipsychotics suspected to have NMS [41]. The prospective study also displayed a high prevalence (12.1%) of NMS/MC [12]. Consistently, the occurrence rate of malignant forms in the present catatonia cohort was 16.0%. Another life-threatening condition in catatonia was the presence of severe medical complications [5]. Our catatonic patients were found to have more deep venous thrombosis, which might be why anticoagulant therapies have been recommended for prevention [42]. The high risk of complications might partially be related to the long-time immobile state and the failure to improve symptoms in a timely manner.

Several factors have been found to be closely associated with the pathogenesis of catatonia including gamma-aminobutyric-acid (GABA)-ergic and glutamatergic dysregulation [11], which was supported by the efficacy of benzodiazepines, the GABA-A receptor agonists, and NMDAR antagonists for the treatment of catatonia. In addition, the activation of the innate immune system, and adaptive immunity by the downstream effects of specific actions on extracellular antigens are also potential related factors [11]. The nosological changes in the DSM-5 [1] and ICD-11 [2] improved the recognition of catatonia due to multiple general medical conditions, indicating the core of etiological treatment in secondary catatonia. Thus, immunotherapy should be fundamental treatment for catatonia due to anti-NMDAR encephalitis, and the simultaneous management of specific treatment (such as benzodiazepines or ECT) and prevention of potential complications may be optimal. Similarly, a prospective study revealed that catatonic syndrome in patients with anti-NMDAR encephalitis was ameliorated by immunotherapy but not lorazepam, and 60% (3/5) of patients achieved only mild relief with ECT [12]. The low response to benzodiazepines may suggest variable underlying mechanisms of catatonia in anti-NMDAR encephalitis. In our cohort, 68.0% of catatonic patients received benzodiazepines, yet it is difficult to judge efficacy in a noncontrolled study. Unfortunately, we were also not able to observe the efficacy of ECT because of its unavailability at our centre. A review concluded that the application of atypical antipsychotics may alleviate the symptoms of non-MC and manic or psychotic episodes. In this cohort, atypical antipsychotics were frequently (92.0%) prescribed in the catatonia group and helped relieve agitation before immunotherapy in 25.0% (6/24). Approximately 42.1% (8/19) of catatonic patients failed to recover from psychiatric symptoms until immunotherapy. Our data suggested the critical value of immunotherapy and the potential positive efficacy of the combined regimen, which was recommended in a previous review [17, 43]. It is worth noting that antipsychotics were withdrawn in three catatonic patients after the aggravation to malignant forms of catatonia in our study, illustrating that the antipsychotics should be administered with caution.

In our study, we found that the catatonia group had higher mRS scores at admission and more ICU admissions, implying that catatonia may either be a marker or a result of a severe disease condition in the acute phase. Considering the causative hypothesis of catatonia, such symptoms might refer to more severe glutamatergic hypofunction in anti-NMDAR encephalitis. The long-term outcomes evaluated by the mRS and CASE were all good, and few differences were observed between the two groups, further indicating that severe glutamatergic dysregulation was immune-related and could be successfully remediated by timely immunotherapy. It has been reported that paediatric autoimmune encephalopathy patients with catatonia suffered more relapses [15]. We found a similar result in adult catatonic anti-NMDAR encephalitis patients. Why and how catatonia is related to relapses remain unknown. Consistent immunotherapy and symptomatic treatment are recommended in these patients.

Anxiety is frequently combined with catatonia [44–46], and catatonic psychiatric inpatients experienced more severe anxiety and affective symptoms than noncatatonic patients at the ictal stage [47]. It is quite interesting to follow up the long-term psychiatric outcomes in patients with anti-NMDAR encephalitis. Our catatonic patients scored poor on the GAD-7 and NPI at follow-up, adding evidence for a long-term relationship between mental behaviour and catatonia in anti-NMDAR encephalitis. However, both the mRS and CASE lack assessments of affective symptoms and catatonic symptoms. Given that, the improvement of long-term global neuropsychiatric evaluation is necessary, including comprehensive assessment combining scales for affective symptoms and specific scale for catatonia (such as the Bush-Francis Catatonia Rating Scale).

## Limitations

Several limitations of this study should be acknowledged. First, our study included only adult patients, and the results do not necessarily apply to younger patients. Second, our study was retrospective, and we lost contact with some patients during follow-up, leading to bias. Third, the sample size of our single-centre study was small, and the statistical analysis was exploratory without Bonferroni correction between comparisons. Considering the limitations of the study, further large-scale and multicentre prospective studies are needed.

## Conclusions

Our study revealed that the characteristics of catatonia in anti-NMDAR encephalitis are heterogeneous. Despite more neuropsychiatric and medical complications in the acute phase, catatonic patients with anti-NMDAR encephalitis can achieve favourable long-term outcomes with prompt immunotherapy initiation. Over the long term, patients with anti-NMDAR encephalitis with catatonia tend to suffer from more relapses and psychiatric problems than those without catatonia.

## Supplementary Information

**Additional file 1: Supplementary Table S1.** Outcomes of patients assessed by mRS and CASE at follow-up.

## Data Availability

The datasets analysed during the current study are available from the corresponding author on reasonable request.

## Abbreviations

NMDAR: N-methyl-D-aspartate receptor
mRS: Modified Rankin scale
CASE: Clinical Assessment Scale for Autoimmune Encephalitis
NPI: Neuropsychiatric Inventory
PHQ-9: Patient Health Questionnaire-9
GAD-7: 7-item Generalized Anxiety Disorder Questionnaire
CSF: Cerebrospinal fluid
ICU: Intensive care unit
DSM-5: Diagnostic and Statistical Manual of Mental Disorders, Fifth Edition
ICD-11: 11th revision of the International Classification of Diseases
CSF: Cerebrospinal fluid
BFCSI: Bush-Francis Catatonia Screening Instrument
MC: Malignant catatonia
NMS: Neuroleptic malignant syndrome
MRI: Magnetic resonance imaging
EEG: Electroencephalograpy
IQRs: Interquartile ranges
ECT: Electroconvulsive therapy
GABA: Gamma-aminobutyric-acid
