# Supplementary material for: Catatonia in adult anti-NMDAR encephalitis: an observational cohort study
Source: BMC Psychiatry. 2023 Feb 7;23:94. doi: 10.1186/s12888-022-04505-x (PMC9903498; doi:10.1186/s12888-022-04505-x)
Supplement: Supplementary file 1 — Additional file 1: Supplementary Table S1. Outcomes of patients assessed by mRS and CASE at follow-up. [file 12888_2022_4505_MOESM1_ESM.doc]

Supplementary Table S1 Outcomes of patients assessed by mRS and CASE at follow-up

| **Outcomes** | **All** | **Non-catatonia** | **Catatonia** | **p-value^*^** |
| --- | --- | --- | --- | --- |
| At 6-month follow-up (n=66) |  |  |  |  |
| mRS scores, median (IQRs) | 1(0-3) | 1(0-3) | 2(0-3) | 0.535 |
| mRS≥3 , n (%) | 20(30.3) | 12(27.9) | 8(34.8) | 0.562 |
| CASE scores, median (IQRs) | 2(1-5) | 2(1-4) | 4(1-6) | 0.226 |
| CASE ≥ 4, n (%) | 25(37.9) | 13(30.2) | 12(52.2) | 0.08 |
| At 12-month follow-up (n=61) |  |  |  |  |
| mRS scores, median (IQRs) | 1(0-2) | 1(0-2) | 1(0-2) | 0.886 |
| mRS≥3 , n (%) | 10(16.4) | 8(20.5) | 2(9.1) | 0.425 |
| CASE scores, median (IQRs) | 1(1-3) | 1(1-3) | 1.5(1-3) | 0.504 |
| CASE ≥ 4, n (%) | 10(16.4) | 6(15.4) | 4(18.2) | >0.999 |
| At 24-month follow-up (n=58) |  |  |  |  |
| mRS scores, median (IQRs) | 0(0-2) | 1(0-1.5) | 0(0-2) | 0.764 |
| mRS≥3 , n (%) | 9(15.5) | 7(18.9) | 2(9.5) | 0.567 |
| CASE scores, median (IQRs) | 1(1-3) | 1(1-3) | 1(1-3) | 0.86 |
| CASE ≥ 4, n (%) | 11(19.0) | 7(18.9) | 4(19.0) | >0.999 |

*P-value less than 0.05 is of significance.
